# Mercury in Hair Is Inversely Related to Disease Associated Damage in Systemic Lupus Erythematosus

**DOI:** 10.3390/ijerph13010075

**Published:** 2015-12-24

**Authors:** William Crowe, Leanne Doherty, Gene Watson, David Armstrong, Elisabeth Ball, Pamela Magee, Philip Allsopp, Aubrey Bell, J. J. Strain, Emeir McSorley

**Affiliations:** 1 Northern Ireland Centre for Food & Health (NICHE), University of Ulster, Coleraine BT52 1SA, Northern Ireland; crowe-w@email.ulster.ac.uk (W.C.); lc.doherty@ulster.ac.uk (L.D.); pj.magee@ulster.ac.uk (P.M.); pj.allsopp@ulster.ac.uk (P.A.); jj.strain@ulster.ac.uk (J.J.S.); 2 Departments of Dentistry, Environmental Medicine, and Pharmacology and Physiology, University of Rochester School of Medicine and Dentistry, Rochester, NY 14642, USA; watson@urmc.rochester.edu; 3 Department of Rheumatology, Altnagelvin Area Hospital, Glenshane Road, Londonderry BT47 6SB, Northern Ireland; oswald17727@hotmail.com; 4 Department of Rheumatology, Musgrave Park Hospital, Stockman’s Lane, Belfast BT9 7JB, Northern Ireland; elisabethball21@googlemail.com (E.B.); aubreybell05@aol.com (A.B.)

**Keywords:** mercury, lupus, disease activity

## Abstract

Systemic lupus erythematosus (SLE) is an autoimmune inflammatory disease, and environmental factors are proposed to exacerbate existing symptoms. One such environmental factor is mercury. The aim of this study was to investigate the relationship between exposure to mercury (Hg) and disease activity and disease associated damage in Total Hg concentrations in hair and urine were measured in 52 SLE patients. Dental amalgams were quantified. Disease activity was assessed using three indexes including the British Isles Lupus Assessment Group Index (BILAG). Disease associated damage was measured using the Systemic Lupus International Collaborating Clinics/American College of Rheumatology SLICC/ACR Damage Index. Pearson’s correlation identified a significant negative correlation between hair Hg and BILAG (*r* = −0.323, *p* = 0.029) and SLICC/ACR (*r* = −0.377, *p* = 0.038). Multiple regression analysis identified hair Hg as a significant predictor of disease associated damage as determined by SLICC/ACR (β = −0.366, 95% confidence interval (CI): −1.769, −0.155 *p* = 0.019). Urinary Hg was not related to disease activity or damage. Fish consumption is the primary route of MeHg exposure in humans and the inverse association of hair Hg with disease activity observed here might be explained by the anti-inflammatory effects of n-3 long chain polyunsaturated fatty acids also found in fish.

## 1. Introduction

Identification of environmental risk factors that stimulate the development of systemic lupus erythematosus (SLE) in genetically susceptible individuals has been the focus of considerable research [1]. The potentially modifiable nature of some of these environmental factors makes this a particularly attractive area of research and presents an opportunity to impact lupus disease activity and subsequent morbidity. Mercury (Hg) is an environmental factor that has been suggested to be associated with the development of SLE [2], albeit the evidence is limited [3].

Hg exists as elemental Hg, inorganic (iHg) or organic (e.g., MeHg) compounds. Humans encounter elemental Hg primarily through inhalation of Hg vapour (Hg^0^) from dental amalgams or through exposure to atmospheric Hg^0^ directly (industrial emissions) [4]. Most inhaled Hg^0^ is absorbed in the lungs and subsequently converted to iHg within the body. Environmental exposure to iHg occurs principally from ingested (non-fish) food, with minor contributions from fish consumption and drinking water. Methylation of iHg to methylmercury (MeHg) by aquatic bacteria and subsequent bio magnification up the aquatic food chain, results in the bioaccumulation of organic MeHg in fish [5]. Fish consumption is the primary route of MeHg exposure in humans. The US environmental protection agency (EPA) reports a reference dose (RfD) level for methylmercury of 0.1 μg per kilogram of body weight per day [6]. The mean hair Hg concentration in a cohort of adult American females in the general population was 0.47 ppm [7] and typical urine Hg levels in an American male population that had amalgams were 3.1 ug/L [8]. In some areas of high exposure to industrial pollution (*i.e.*, the mercury poisoning that occurred at Minamata), hair Hg concentrations have reached concentrations of 705 ppm [9]. The no observed adverse effect level for hair mercury is 50 ppm, whilst for pregnant individuals this is lowered to 10 ppm [10].

Both *in vitro* and *in vivo* studies have demonstrated the ability of various Hg species to cause damage and dysfunction to a number of physiological systems including the immune system. *In vitro* studies report that both iHg and MeHg exposures result in decreased cell proliferation capacity [11], dysregulation of pro- and anti-inflammatory cytokine balance [12,13,14] and increased lymphocyte apoptosis [15]. Changes observed in cytokine production have been suggested to drive responses of autoreactive T cells towards the development of autoimmunity [16,17]. Animal studies consistently demonstrate that both iHg and MeHg exposure induces systemic autoimmunity in those who are genetically susceptible [18,19,20,21] and exacerbates autoimmune symptoms in animal models which spontaneously develop lupus like disease [22]. Furthermore, studies report acceleration in the development of autoantibodies and immune complex (IC) deposits following organic Hg (oHg) treatment in models of idiopathic autoimmunity [23].

Epidemiological studies have reported that increased exposure to Hg^0^, owing to an individual’s exposure to industrial Hg^0^ pollution, is linked with an increased prevalence of SLE [2,24,25]. The immunotoxic effects of chronic low level exposure to Hg^0^ in humans has been postulated to be associated with an increased risk of developing lupus [26] and scleroderma [27]. Occupational exposure of Hg^0^ has been associated with increased concentrations of autoimmune anti-nuclear autoantibodies by some [28,29] whereas others have not observed any association [30,31,32].

Autoimmune/inflammatory syndrome induced by adjuvants (ASIA) describes an autoimmune/inflammatory disease which develops in response to exposure to a component that contains an adjuvant [33]. Although limited, research to date suggests that Hg (both MeHg and Hg^0^) exposure in certain individuals elicits a syndrome similar to ASIA [34]. However, no study has investigated Hg exposure in SLE using biomarkers and clinical endpoints. Therefore, the aim of this study was to investigate the relationship between Hg exposure measured in hair (biomarker of MeHg exposure), urine (biomarker of Hg^0^ exposure), and dental amalgam status (indirect biomarker of Hg^0^ exposure) and clinically determined disease activity and disease associated damage in SLE patients.

## 2. Materials and Methods

### 2.1. Study Design

Participants were identified through rheumatology clinics in the Belfast Health and Social Care Trust (BHSCT) and Western Health and Social Care Trust (WHSCT), Northern Ireland. Participants were recruited as part of a larger study that assessed the relationship between vitamin D status and disease activity [35]. All participants met the criteria for diagnosis of SLE as defined by the American College of Rheumatology (ACR) criteria [36]. Ethical approval was obtained from the Office of Research Governance Northern Ireland (10/NIR02/43) and all participants provided written informed consent. The research adhered to the standards outlined in the Declaration of Helsinki 1975 (revised Hong Kong 1989).

### 2.2. Clinical Assessment

The assessment of disease activity and damage was performed by one of two consultant rheumatologists experienced in the use of clinical assessment tools in the research setting. Participants were evaluated for disease activity using the British Isles Lupus Assessment Group Index (BILAG), Systemic Lupus Activity Measure (SLAM), the revised Safety of Estrogen in Lupus Erythematosus National Assessment (SELENA) version of the Systemic Lupus Erythematosus Disease Activity Index (SLEDAI), and disease associated damage was assessed using the SLICC/ACR damage index. All disease indices and samples were collected on the same day.

BILAG uses subjective and objective measures to assess the extent to which an organ system is contributing to disease activity over the previous 4 weeks. Each organ system is assigned a grade based on disease activity [37]. A cumulative numerical BILAG score was calculated using the collective grades from each organ system [37]. SLICC/ACR+ determines disease associated damage that has occurred after diagnosis of SLE. Symptoms are required to be present for at least 6 months. The score range is 0–47 [38]. SLAM evaluates 11 organ systems and considers 30 variables, a score of 7+ is considered clinically significant and distinguishes active disease from periods of remission and parameters spanning the previous 30 days are measured [39,40]. SELENA-SLEDAI considers symptoms over the previous 30 days and contains 3 components that evaluate new disease activity, the deterioration of an existing ailment, increases or decreases in medication and the physician’s global assessment visual analogue scale score. A score of 4+ indicates active disease [41]. Disease activity measures have been shown to correlate with each other in clinical trials, but the precise measures will vary within and between patients. In using all 3 SLE disease activity measures along with a measure of damage we will be able to investigate recent and cumulative effects of Hg exposure.

#### Serological Measures

Blood samples in serum separator tubes were centrifuged for 15 min at 3000 *g*. An aliquot of serum was analysed for anti-double stranded DNA antibodies, C3 and C4 at the Royal Victoria Hospitals Belfast immunology labs which is a member of the UKNEQAS scheme for nuclear and related antigens. Venous blood was collected into a trisodium citrate tube for the measurement of erythrocyte sedimentation rate (ESR). ESR was measured using the Westergren method. The fluorescence enzyme linked immunoassay (Phadia, Immunocap 250) was utilised for detection of anti-dsDNA. Plasmid dsDNA was coated to a solid phase support. Serum samples were diluted 1/10 and incubated for 30 min. Secondary antibodies and washing steps were automated. C3 and C4 were quantified by rate nephleometry (Beckman Image 8000).

### 2.3. Urine and Hair Collection

Hair samples were taken from participants who consented to having their hair cut. Approximately 50–100 strands of hair were held between the finger and thumb near the base of the scalp of each patient. The hair was then clamped with a haemostat and cut with surgical scissors as close to the scalp as possible by the researcher. Samples were then tied firmly with cotton thread 1–2 cm from the cut end before the haemostat was released. Hair samples were then placed in a labelled envelope to be stored at room temperature. The closest 1 cm of hair to the head was then analysed using cold vapour atomic absorption spectrometry for total Hg. A 30 mL fasted urine sample (second morning void) was collected and stored at −80 °C and subsequently analysed for total mercury using atomic fluorescence spectrometry.

### 2.4. Anthropometric Assessment

Body weight (kg) was measured to the nearest 0.1 kg using portable scales (Seca; Brosch Direct Ltd., Peterborough, UK). Height (m) was measured to the nearest 0.1 cm using a stadiometer (SECA, Model 220, Hamburg, Germany). Body mass index (BMI) was calculated as weight (kg) divided by height squared (m^2^).

### 2.5. Urinary and Hair Mercury Assessment

Hair and urine samples were shipped to the University of Rochester, Rochester, NY, USA, for Hg analysis. Hair Hg concentrations were determined using cold vapour atomic absorption and the “Magos” reagents and method [42]. MeHg is the predominant form of Hg that exists in hair. Hg below the limit of detection (<0.75 ppm) was imputed as 0.75/2 ppm [43]. Urine samples were assessed for total Hg concentrations (ng/mL) by atomic fluorescence spectrometry (PSA 10.035 Millennium Merlin, PS Analytical Ltd., Kent, United Kingdom) by methods used previously [44]. Assessment of Hg within the urine reflects inorganic Hg, primarily from dental amalgams [9]. The accuracy of the methods was assessed in all analyses by including standard reference materials for hair (IAEA-085 and IAEA-086, International Atomic Energy Agency) and urine (Seronorm, SERO AS, Stasjonsvn, Norway). The Mercury Laboratory at the University of Rochester participates in external quality assessment for mercury analyses through participation in the Interlaboratory Comparison Program for Metals in Biologic Matrices (Institut National de Santé Publique, Quebec, Canada). The University of Rochester Mercury Analytical Laboratory served as a Reference Laboratory for analysis of hair for the recent quality assessment of laboratories within the transnational COPHES/DEMOCOPHES project [45]. Urinary Hg concentrations were normalised by adjusting with creatinine concentration (μmol/L) as measured by the ILAB 650 (SpA, Milan, Italy). The intra assay precision coefficient of variance (CV) for creatinine was <1.7%.

### 2.6. Quantification of Dental Amalgams

Participants were provided with a mirror in order to self-report the quantity of their own dental amalgams. All amalgams were given a score of 1 regardless of their size or location. Patients also provided information on the removal and replacement of any previous dental amalgams.

### 2.7. Statistical Analysis

Statistical analysis was undertaken using IBM^®^ SPSS^®^ Statistics v20. Similar to previous studies the distribution of Hg concentrations (hair and urine) was skewed [46], while disease indexes and serological measures were also skewed and thus these parameters were log transformed for plotting and analysis. Results are presented using the mean (±SD) on their natural scale.

The primary aim was to investigate for associations between hair Hg or urinary Hg and measures of disease activity and disease associated damage. Correlation analysis was used to determine associations between hair Hg and disease activity and disease associated damage, and also between urinary Hg concentration and disease activity and disease associated damage. Pearson’s correlation was used to assess the relationship between each of the disease activity and disease associated damage indexes. Standard multiple regression analysis was carried out to determine the strength of the relationship between Hg in both hair and urine, and disease activity and disease associated damage. Model 1 was statistically adjusted for the potential influence of age. Model 2 was adjusted for age and BMI. Standard multiple regression analysis was used to determine the relationship between the serological measures (anti-dsDNA, C3, C4, and ESR) and mercury exposure, whilst controlling for age and BMI.

Secondary analysis was carried out to investigate the association between dental amalgam count and disease activity and disease associated damage and to determine if there is a difference in Hg concentrations (hair and urine) between those with active disease and those with inactive disease. Independent *t*-tests were used to test for differences in mean hair Hg and urinary Hg concentration between those with active and inactive disease.

## 3. Results

### 3.1. Study Population

The patient’s characteristics are detailed in Table 1. The mean (±SD) age of the 52 SLE patients in this study was 48 (±13.19) years with a mean BMI of 27 (±4.96) kg/m^2^. Some 94.2% of patients were female. The mean (±SD) disease activity and disease associated damage scores were BILAG 6.1 (±6.29), SLAM 5.66 (±2.39), SELENA SLEDAI 4.46 (±4) and SLICC/ACR 0.96 (±1.12). SLAM identified 13 out of 52 (25%) of patients to be currently suffering from a disease flare (score > 7), whilst SELENA SLEDAI identified one patient (score > 6). Mean (±SD) hair and urine Hg concentrations were 1.50 (±1.55) ppm and 1.1 (±1.24) ng/g creatinine, respectively. The mean (±SD) number of dental amalgams was 4.9 (±4.6) surfaces and the average number removed was self-reported to be 0.8 (±1.9), bringing the total amalgams ever installed to an estimated average of 5.6 (±5.4) surfaces. Pearson’s correlation showed that BILAG correlated with SLAM and SLICC (*p* ≤ 0.001, *p* = 0.010, respectively) SLAM correlated with SLICC (*p* = 0.020), whilst SELENA SLEDAI did not correlate with any other index.

**Table 1:** Characteristics of systemic lupus erythematosus patients.

	Mean (±SD)
**N =**	52
**Age**	48 (±13.19)
**Gender—Female (%)**	50 (96.2%)
**BMI**	27 (±4.96)
**Smoker, *n* (%)**	9 (17.3%)
**Taking immunosuppresants, *n* (%)**	7 (13.5%)
**Taking steroids, *n* (%)**	12 (23.1%)
**Anti-DsDNA antibody iu/mL**	23.2 (±42.7)
**Anti-DsDNA positive, *n* (%)**	16 (30.8%)
**ESR mm/h**	15.2 (±18.04)
**C3 g/L**	1.09 (±0.31)
**C4 g/L**	0.21 (±0.14)
**SLAM**	5.66 (±2.39)
**SELENA SLEDAI**	4.46 (±4)
**BILAG**	6.1 (±6.29)
**SLICC/ACR**	0.96 (±1.12)
**Neurological involvement, *n* (%)**	5 (9.6%)
**Renal involvement, *n* (%)**	4 (7.7%)
**Urine Hg ng/g creatinine**	1.1 (±1.24)
**Hair Hg ppm**	1.5 (±1.55)
**Dental amalgams**	4.9 (±4.6)

Anti-DsDNA antibody: anti double stranded DNA antibody; BILAG: British Isles Lupus Assessment Group index; BMI: Body mass index; C3: Complement 3; C4: Complement 4; ESR: erythrocyte sedimentation rate; Hg: mercury; SELENA SLEDAI: Safety of Estrogen in Lupus Erythematosus National Assessment Systemic Lupus Activity Index; SLAM: Systemic Lupus Activity Measure; SLE: Systemic Lupus Erythematosus; SLICC/ACR: Systemic Lupus International Collaborative Clinics/American College of Rheumatology.

### 3.2. Univariate Correlations

A significant negative correlation was observed between hair Hg and BILAG (*r* = −0.323; *p* = 0.029) but not with SLAM (*r* = −0.25; *p* = 0.093) or SELENA SLEDAI (*r* = 0.300; *p* = 0.843). Hair Hg was negatively correlated with SLICC ACR (*r* = −0.377; *p* = 0.038) (Table 2). Urinary Hg did not significantly correlate with any measure of disease activity or disease associated damage.

Secondary analysis found no relationship between the quantity of dental amalgams and any measure of disease activity or disease associated damage. Number of dental amalgams present correlated positively with urine Hg concentration (*r* = 0.541, *p* ≤ 0.001) but not with hair Hg (Table 3).

### 3.3. Multiple Regression Analysis

The degree to which Hg predicts disease activity and disease associated damage was estimated by standard multiple regression (Table 3). Table 2 shows a significant inverse correlation between hair Hg and BILAG; however, as shown in Table 3, this association was no longer significant after controlling for age and BMI. Hair Hg was not a significant predictor of any other measure of disease activity.

**Table 2:** Correlation between markers of Hg exposure and disease activity and damage in SLE (*n* = 52).

Markers of Hg Exposure	BILAG		SLAM		SELENA SLEDAI		SLICC ACR	
	r	*p*	r	*p*	r	*p*	r	*p*
**Hair Hg**	−0.323	0.029 *	−0.251	0.093	0.105	0.843	−0.377	0.038 *
**Urine Hg**	−0.081	0.592	−0.078	0.605	−0.023	0.895	0.182	0.226
**Dental Amalgams**	0.222	0.274	0.253	0.086	−0.218	0.136	−0.032	0.830

BILAG; British Isles Lupus Assessment Group index; Hg: mercury; SELENA SLEDAI: Safety of Estrogen in Lupus Erythematosus National Assessment Systemic Lupus Activity Index; SLAM: Systemic Lupus Activity Measure; SLE: Systemic Lupus Erythematosus; SLICC/ACR: Systemic Lupus International Collaborative Clinics/American College of Rheumatology. Statistical analysis was performed using Pearsons correlation. * denotes significance *p* < 0.05.

**Table 3:** The relationship between urine and hair Hg and measures of disease activity and damage in SLE (*n* = 52).

Model	Predictor Variables	BILAG				SLAM			
		Model *R*^2^	*p* Value	(95% CI)	Beta	Model *R*^2^	*p* Value	(95% CI)	Beta
1	Hair Hg	0.111	0.062	−0.189, 0.105	−0.088	0.065	0.096	−3.250, 0.273	−0.088
	Age			0.569	−0.189, 0.105		−0.088	0.752	−0.048, 0.067	0.05
2	Hair Hg	0.111	0.066	−.884, 0.293	−0.294	0.127	0.131	−3.057, 0.411	−0.238
	Age			0.571	−0.191, 0.107		−0.088	0.697	−0.45, 0.067	0.06
	BMI			0.928	−0.378, 0.345		−0.013	0.091	−0.020, 0.253	0.252
1a	Urine Hg	0.02	0.621	−6.250, 3.774	−0.188	0.007	0.614	−2.398, 1.434	−0.12
	Age			0.449	−0.200, 0.090		−0.116	0.89	−0.59, 0.052	−0.12
2a	Urine Hg	0.02	0.629	−6.399, 3.910	−0.076	0.057	0.808	−2.152, 1.687	−0.037
	Age			0.323	−0.203, 0.092		−0.116	0.974	−0.056, 0.054	−0.021
	BMI			0.988	−0.401, 0.395		−0.002	0.141	−0.038, 0.258	0.229
		**SELENA SLEDAI**				**SLICC ACR**			
1	Hair Hg	0.031	0.586	−2.242, 4.078	−0.267	0.021	0.019 *	−1.769, −0.155	−0.366
	Age			0.258	−0.159, 0.044		−0.186	0.263	−0.012, 0.041	0.173
2	Hair Hg	0.057	0.506	−2.115, 4.223	−0.238	0.147	0.015 *	−1.823, −0.204	−0.385
	Age			0.28	−0.157, 0.047		0.177	0.281	−0.012, 0.040	0.166
	BMI			0.301	−0.120, 0.378		0.16	0.256	−0.100, 0.027	−0.165
1a	Urine Hg	0.264	0.821	−1.126, 1.412	0.33	0.182	0.234	−0.351, 1.4	0.184
	Age			0.081	−0.069, 0.004		−0.263	0.939	−0.024, 0.026	0.012
2a	Urine Hg	0.266	0.791	−1.132, 1.477	0.04	0.203	0.288	−0.418, 1.375	0.165
	Age			0.088	−0.070, 0.005		−0.26	0.973	−0.25, 0.026	0.005
	BMI			0.797	−0.088, 0.114		0.039	0.558	−0.089, 0.049	−0.091

BILAG: British Isles Lupus Assessment Group index; BMI: body mass index; CI: confidence intervals; Hg: mercury; SELENA SLEDAI: Safety of Estrogen in Lupus Erythematosus National Assessment; Systemic Lupus Activity Index; SLAM: Systemic Lupus Activity Measure; SLE: Systemic Lupus Erythematosus; SLICC/ACR: Systemic Lupus International Collaborative Clinics/American College of Rheumatology. Statistical analysis was performed using standard multiple regression. * denotes significance *p* < 0.05.

Hair Hg was not a significant predictor of any measure of disease activity. Hair Hg was a significant inverse predictor of disease associated damage (β = −0.366, *p* = 0.019; 95% confidence interval (CI): −1.769, −0.155) and explained 2% of the variation in disease associated damage as measured by SLICC ACR. Further analysis controlling for BMI identified that hair Hg explained ≈15% of the variation in disease associated damage (β = −0.385, *p* = 0.015, 95% CI: −1.823, −0.204). Regression analysis identified that urinary Hg did not significantly predict disease activity or disease associated damage.

Neither Hair Hg nor urinary mercury were significantly related to serological markers, except ESR, which was inversely related to hair Hg (β = −0.297, *p* = 0.05, 95% CI: −0.531, −0.001). When controlling for age and BMI no relationship existed between any serological markers and indices of Hg exposure.

### 3.4. Hg Concentrations in Those with Active Disease versus Inactive

**Figure 1:**
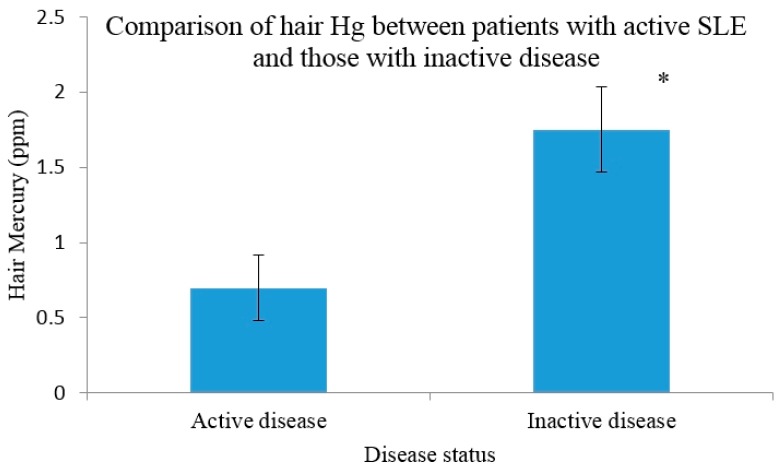
Hair mercury levels in systemic lupus erythematosus patients stratified by disease activity (SLAM) using 7+ as a cut off. Error bars indicate the SEM. * denotes significance *p* < 0.05.

Participants were classified as active or inactive according to each disease activity measure. As shown in Figure 1, those with an active disease status according to SLAM (*n* = 14) had a significantly lower hair Hg (0.7 ppm) than those who had an inactive disease (*n* = 38) (1.75 ppm, *p* = 0.009). There was no significant difference between hair Hg in those with active disease (*n* = 6) (1.2 ppm) compared to those with inactive disease (*n* = 46) (1.69 ppm) as determined by BILAG, albeit this approached significance (*p* = 0.062). SELENA SLEDAI identified one patient as suffering from active disease; therefore no analysis was completed. No differences in urinary Hg concentrations were observed between those with active disease and those who were classified as inactive (Figure 1).

## 4. Discussion

A significant inverse relationship was observed between hair Hg and disease associated damage as measured by SLICC/ACR, although we observed no relationship between hair Hg, and disease activity. Furthermore no association was observed between disease activity or disease associated damage and urinary Hg or number of dental amalgams. Those with inactive disease had significantly higher hair Hg concentrations than those with active disease as determined by SLAM.

Results from this study would indicate that higher concentrations of Hg in the hair are predictive of decreased disease associated damage in SLE. Although not significant the results are suggestive of a trend between disease activity (BILAG & SLAM) and hair Hg and are supported by the finding that patients in remission have significantly higher hair Hg than patients with active disease. BILAG is the most detailed assessment of disease activity followed by SLAM and both assess changes deemed to be clinically significant over the previous month.

Hair Hg consists mostly of MeHg and represents the previous month’s exposure to MeHg. All fish bio-accumulate MeHg and dietary fish consumption is the principal exposure route for MeHg in humans [5]. Fish are also a rich source of long chain n-3 polyunsaturated fatty acids (LCPUFA) and previous work by us and others has identified LCPUFA ingestion as having a positive influence on disease activity in SLE [47,48]. Furthermore, research investigating the relationship between healthy maternal MeHg exposure and markers of neurological child development has reported an inverse relationship between MeHg and cognitive function [49], which is attributed to the beneficial effects of LCPUFAs from fish consumption [50]. The anti-inflammatory properties of n-3 LCPUFA result from their ability to act as a substrate for the production of anti-inflammatory eicosanoids. The n-3 LCPUFA can also modify inflammatory gene expression via peroxisome proliferator-activated receptors (PPARs) to skew cytokine profiles towards an anti-inflammatory state [51]. Fish also contain α-tocopherol and carotenoids, which are potent scavengers of free radicals. In addition, fish are a major contributor to selenium intake and selenium has been linked with a range of health benefits including improved immune function, decreased risk of cardiovascular disease and protection against certain forms of cancer [52]. Therefore, in this study, it is plausible that hair Hg is acting as a surrogate marker for fish consumption and the nutrients present in fish are eliciting a beneficial effect on the SLE patients health. This possibility is supported by findings from the National Health and Nutrition Examination Survey (NHANES) 1999–2004, which reported that Hg exposure was associated with ANA positivity and adjusting for n-3 fatty acid status increased the magnitude of the association [53].

Another possible explanation for the apparent protective effect of a higher hair MeHg concentration on disease associated damage observed in this study is that MeHg is acting as an immunosuppressive [20]. Previous animal and *ex vivo* studies have reported MeHg to reduce immunoglobulin response following a challenge with an immunogen [54], as well as decreasing proliferation response [15,55]. Therefore, it is plausible that MeHg may be acting as an immunosuppressant and reducing disease associated damage.

Autoimmune diseases, including SLE, have been linked with exposure to dental amalgams [56]; yet the scientific evidence for such a link is lacking. Similar to others, we observed that urinary Hg correlated with the number of dental amalgams in the SLE patients [57], and thereby suggesting that urine Hg is a good biomarker of Hg^0^ exposure in this population. Urinary Hg concentrations were relatively low (1.1 ng/g creatinine) in comparison to occupationally exposed cohorts (3.67 ng/mL (unadjusted for creatinine)) [46] but higher than a non-occupationally exposed healthy cohort (0.46 ng/g creatinine) [58]. The absence of an association between urinary Hg and disease activity or disease associated damage suggests that Hg^0^ exposure, at the low concentrations observed in this study, is not related to disease status. Similarly no association exists between disease outcomes and the quantity of dental amalgams present. These findings may be owing to the low levels of iHg exposure, although previous research has suggested that biologically relevant doses of HgCl_2_ can cause autoimmunity in predisposed animals [22]. The majority of animal studies conducted to date have used concentrations higher than humans would normally encounter [18,19,22]. To our knowledge this study is the first to analyse Hg exposure in SLE patients and the finding that neither MeHg or Hg^0^ exposure impacts negatively on disease outcomes has important implications for public health advice given to patients. These findings contribute important information regarding the safety of dental amalgam use, particularly in patients with autoimmunity.

A limitation of this study is that we have no measure of fish intake or fatty acid profile which would aid in the interpretation of these results. Habitual Hg exposure (MeHg and Hg^0^) is unknown as the sample was taken at one time point only. Speciation of hair samples would further improve the interpretation of these results. The use of hair as a marker of MeHg exposure is imperfect as total hair Hg does not consist entirely of MeHg. Hair Hg may contain ethyl Hg encountered in vaccines [59] but we did not record any detail on vaccine use in this study. Vaccines have been implicated in the development of ASIA syndrome [33]. As hair Hg in this study was relatively low it is unclear how exposure to high dose MeHg would interact with disease activity and disease associated damage in SLE. An increased sample size would have allowed sub analysis comparing Hg exposure in those with specific disease manifestations such as renal and central nervous system involvement.

## 5. Conclusions

This study provides evidence that low dose Hg^0^ exposure from dental amalgams is not associated with disease activity or disease associated damage in SLE. It is postulated that the inverse correlation observed between hair Hg and disease associated damage is confounded by unmeasured nutritional covariates such as LCPUFA status. Future research would benefit from the collection of dietary data to assess fish intake along with a measure of serum LCPUFA.
